# “That's just typical!” A qualitative video review study on the ecological validity of couples' stress conversations in the lab

**DOI:** 10.3389/fpsyg.2025.1497690

**Published:** 2025-12-02

**Authors:** Katharina Weitkamp, Jasmin Constam, Guy Bodenmann

**Affiliations:** Department of Psychology, Clinical Psychology for Children/Adolescents and Couples/Families, University of Zurich, Zurich, Switzerland

**Keywords:** behavioral observation, couples, ecological validity, dyadic coping, video review task

## Abstract

**Objective:**

For research on dyadic coping—how partners in intimate relationships deal with stress together—video-recorded behavioral observations in a lab are considered to be the best way to study actual coping behavior interactions. To date, little is known about the ecological validity of these laboratory situations. The current study aimed to ask couples directly what they considered to be typical when looking back at a videotaped behavioral interaction sequence in the lab.

**Methods:**

A qualitative video recall approach was used in which interview data were collected. *N* = 117 couples participated in a videotaped stress communication in a lab setting and were subsequently separately interviewed in a video-review task on the typicality of the lab situation and their partner's behavior. Interviews were audiotaped and analyzed using qualitative content analyses.

**Results:**

Participants mostly viewed their partner's behavior as typical. However, the setting and the course of the conversation were viewed as less typical. There were gender and age differences with regards to the ecological validity of lab settings.

**Conclusions and implications:**

Results indicate that the ecological validity of dyadic coping in the laboratory is multidimensional. The lab setting seems comparable to situations where both partners have time and are not stressed.

## Introduction

Reasons for experiencing stress are manifold. Dealing with stress positively in a romantic relationship is viewed as a protective factor for health and wellbeing (Landolt et al., 2023; Weitkamp et al., 2021) as well as relationship satisfaction (Falconier et al., 2015) and stability (Bodenmann and Cina, 2006). A way to deal with these arising stressors together is dyadic coping (DC) as laid out in the Systemic Transactional Model (STM; Bodenmann, 1995, 1997, 2000). In the STM, DC is conceptualized as a process comprising (a) the stress signals of one partner, the perception and interpretation of these signals by the other partner, and their response reactions (ignoring, verbal and non-verbal coping that can be positive or negative), (b) DC depends on the current or cross-situational stable coping resources of both partners (dyadic skills) as well as the current situation or stress constellation and, (c) DC is elicited depending on the partner's current or global motivation, expectations and attitudes toward the relationship (e.g., relationship satisfaction, commitment, etc.) and the partner as well as common goals. DC can take different forms, like supportive, delegated or joint/common DC. DC can be either emotion- and/or problem-focused (Bodenmann, 1995; Bodenmann et al., 2016). Numerous studies have shown that DC is a significant predictor of relationship functioning (see Weitkamp and Bodenmann, 2022 for a current review).

Previous studies on DC have mainly relied on questionnaires, and to a lesser degree on behavioral observations with subsequent video analysis, or diary studies (Weitkamp and Bodenmann, 2022). The current understanding of the importance of stress coping in intimate relationships is therefore mostly based on self-reports. Video/audio recordings of actual DC interaction sequences were utilized only in a limited number of studies, even though these enable direct and objective observation of the dynamic interplay within couple interactions. In these behavioral observation settings, couples were often invited to talk about a stressful experience, to observe stress expression, to listen to their partners, and to engage in actual DC behaviors with the assumption that the presented behavior would be somewhat similar to the behavior of the couple in a natural setting (i.e., in the sense of external or ecological validity; Orne, 1962). Such studies often focused on the use of “we”—language or the use of the first-person plural pronoun (Lyons et al., 1998), behavioral coding of communal coping (Helgeson et al., 2018), or coding of DC-processes (Kuhn et al., 2017, 2018; Meuwly et al., 2012). Observational studies of relationship functioning have been carried out in various areas, for instance on (a) physical health issues, like communal coping in couples affected by type 2 diabetes (Van Vleet et al., 2018), (b) mental health issues, like ways of relational conflict communication interacting with alcohol use disorder (Fairbairn and Cranford, 2016), (c) specific major stressors, like problem-solving and support interactions of couples in the transition to parenthood (Tanner Stapleton and Bradbury, 2012), or personality traits affecting relationship quality, like the role of attachment orientations during couple conflict conversations (Winterheld et al., 2013).

Compared with self-report questionnaires, behavior observations allow for a more objective assessment of couple interactions when systematic coding systems are utilized (Bierhoff and Petermann, 2013). However, artificial laboratory settings likely differ from those found in the couple's everyday interactions at home, but nevertheless allow the collection of a reliable and valid glimpse into the important features of dyadic interactions (Bulling et al., 2023). Whether research using behavioral observation can capture the complex coping processes of couples in a way that is representative of the coping behavior taking place in everyday life remains under-studied.

Since no definition of the “real world” (Hammond, 1998) exists to which behavioral observation data may be compared, the logical source of reference to assess the ecological validity of the laboratory setting and the presented behavioral interactions are the study participants themselves who know about their partner's behavior in their everyday lives. So far, this subjective perspective has been mostly neglected. To our knowledge, the first and only investigation to ask study participants themselves about the representativeness of video-taped couple conversations was by Foster et al. (1997). They examined 239 couples affected by loss of employment who talked about the stress of their job loss in a video-taped in-home conversation setting. After the video-taped discussion, participants separately rated the typicality of their partner's social support and undermining behavior during the video-taped conversation. Even though conversations took place in the couple's homes, less than half of participants rated their partner's social support behavior as the same as usual (42% of women, 46% of men) and over a third rated the social support as more than usual (37% both, women and men). These findings from self-report questionnaire data somewhat challenge the so far untested assumption of the ecological validity of couple conversations in research settings. There is a lack of research investigating the subjective perspective on observational studies in more detail. Additionally, updating this knowledge seems relevant, since observation dynamics and settings may have changed, as well.

Important to note, ecological validity is not a one-dimensional construct (Naumann et al., 2022). Schmuckler (2001) proposed a multimodal analysis of ecological validity, with the dimensions of context, stimuli, and response. Translating this to the dyadic behavior observation setting, context validity would be the transferability of the lab situation to the home, stimulus validity would be the topic of conversation the couple covers in the lab setting, and response validity could be understood as the way the conversation unfolds and how the behavior of both partners is dis-/similar to their everyday conversations at home. This differentiation has not yet been considered when testing the ecological validity of couple behavior observations. Gaining greater insight into ecological validity would ground behavioral couple observations on a firmer footing. It can yield important findings for basic and applied research. To our knowledge, no study to date has examined the ecological validity of behavioral observations among couples in sufficient detail or has incorporated the participants' own perspectives, even though these participants are best positioned to assess ecological validity. Consequently, it is important to fill this knowledge gap.

Thus, the aims of the current study were to investigate (a) what study participants themselves view as similar or different when looking at their laboratory behavioral interaction sequence compared with their day-to-day conversations at home, and (b) whether overall, context (lab situation), stimulus (conversation topic), and response (partner behavior/couple conversation) are viewed as typical to their day-to-day lives. Both qualitative and quantitative/mixed-methods analyses were conducted. Specifically, we examined the following research questions using qualitative analysis:

1. What is described as un-/typical by research participants, when reviewing their DC interaction sequence in the lab?
2. How representative of their day-to-day DC is the conversation in the lab for study participants?

In addition, the mixed-methods analyses were exploratively conducted to examine whether participants with certain demographics (gender and age) would view the lab experience as more/less typical than others.

## Methods

### Procedure

The current project was part of a larger longitudinal multi-method observational study that consisted of 10 annual measurement time points (Kerkhoff et al., submitted).^1^ Couples were recruited in 2011 via radio and newspaper advertisements. Since we were interested in general in couple interactions, the sample consisted of non-clinical community-dwelling couples in committed relationships. To be eligible, couples had to be in their current relationship for at least 1 year. Interested couples were informed about the study and after agreeing to participate, they were instructed to independently complete a set of questionnaires. In waves t1–t5 and t10, the questionnaires were followed up by a laboratory session with videotaped couple conversations. Data for the current study are from wave t10.

The lab situation in wave t10 followed a structured procedure of three interaction sequence (each lasting 8 min.), namely one dyadic coping conversation for each partner followed by a conflict conversation, where couples talked about a current internal stressor. For these interactions, couples started together in a room equipped with two comfortable chairs facing each other at an angle so that video cameras were able to tape faces from the front. In total four video cameras recorded the interactions, one wide-angle from the front, one from the back, and one camera directed at each partner. To assess dyadic coping behavior, partners each identified a recent stressor arising outside of the relationship from a list and were then invited by the experimenter to talk about one of these individual external stressors as they normally would at home. It was randomly determined which partner's stressor the couple would focus on first—partner A's or partner B's. These interactions were video-recorded from the above mentioned four different angles. During all interaction sequences the experimenter left the room to give the couple a sense of privacy. Following these three interaction sequences, to capture the subjective experience of the videotaped stress conversations, we used video-stimulated recall, a technique mainly applied to assess psychotherapy processes (Elliott and Shapiro, 1988) or professional-client interactions (Larsen et al., 2008; Paskins et al., 2017). Both partners were guided to separate rooms and separately shown the videos where they talked about their own stress. They viewed the camera perspective facing the partner. Participants were invited to ask for the video to be stopped whenever they wanted to comment on anything in the situation or when their partner's behavior appeared particularly typical, positive, bothering, striking, or noteworthy. After watching 8 min. of the video and commenting, the video was stopped and participants were interviewed by the experimenter in more detail about what they thought was typical or not using a short semi-structured interview guideline. Experimenters were trained graduate students who were not involved in the subsequent data analyses.

All procedures were evaluated and approved by the Ethics Committee of the [University of Zurich]. Participants were informed about the study and gave their written approval. Upon completion of the interactions, couples were debriefed and received approximately 110$.

### Sample

The sample consisted of non-clinical highly stable couples from rural and urban regions of Switzerland. In total, *N* = 368 heterosexual couples filled out questionnaires and took part in videotaped conversations at wave 1. At wave 10, *N* = 119 couples (32.3%) remained to participate in the laboratory session, however, for two couples video files were missing. Thus, the database for the current analyses consisted of *n* = 117 couples (*n* = 234 individuals). Women were on average *M* = 56.83 (*SD* = 15.45) and men *M* = 59.21 (*SD* = 15.42) years old and in the relationship over 20 years (*M* = 21.33; *SD* = 18.23). The majority was married in their first marriage (82% women, 80% men), just over 10% were in their second marriage, less than 8% were unmarried or divorced/widowed in previous relationships. Couples were mostly living together either as a couple (about 91%) or in a shared house with the partner and other people (about 6%) only one man and one woman reported they lived without the partner. About 80% had children and over 38% of women and 64% of men had a college or university degree, see Table 1 for details.

**Table 1:** Sample characteristics.

**Variable**	**Women (*****n*** = **117)**		**Men (*****n*** = **117)**	
	***M***	***SD***	***M***	***SD***
Age	56.83	15.45	59.21	15.42
Relationship duration	21.33	18.23	–	–
	***n***	**%**	***n***	**%**
**Level of education**				
Mandatory school	2	1.7	1	0.9
Vocational training	49	41.9	26	22.2
High school	19	16.2	13	11.1
College/university	45	38.5	75	64.1
15.6-2.2,-1.3242ptNo information	2	1.7	2	1.7
**Marital status**				
Unmarried	4	3.4	5	4.3
Married (1st marriage)	96	82.1	93	79.5
Married (2nd marriage)	12	10.3	15	12.8
Divorced	4	3.4	4	3.4
15.6-2.2,-1.3242ptWidowed	1	0.9	–	–
**Living situation**				
Alone	–	–	1	0.9
With partner	107	91.5	106	90.6
Shared house (with partner)	8	6.8	5	4.3
Shared house (without partner)	1	0.9	–	–
Other	1	0.9	–	–
No information	–	–	5	4.3
Children/step-children (yes)	95	81.2	92	78.6

### Qualitative analyses

The qualitative analysis followed two blocks of analyses: In the first block, we carried out an inductive/deductive qualitative content analysis (Mayring, 2015) looking at what participants described as un/-typical of the laboratory situation. In the second block, we utilized the deductive qualitative analyses method of scaling structuring following Mayring (2015). Here, we used the categories formulated in the first block as the basis to reach a global judgement for each participant whether they viewed the laboratory situation overall as *typical, slightly typical/mixed*, or *untypical*.

#### Categorization of which aspects participants perceive as typical (inductive/deductive analysis)

The qualitative content analyses follows a set of steps that structures and systematizes the analysis (Mayring, 2015). For the analyses, a deductive category grid was developed with the two dimensions *typicality* and *ecological validity*. Typicality was subdivided into three categories: *typical, mixed/slightly typical*, or *untypical*. Ecological validity was subdivided into three dimensions of ecological validity based on Schmuckler (2001): *partner characteristics/partner support* (response), *conversation* (stimulus), and *setting* (context). Within this deductive grid, tentative inductive categories were formulated by the second author based on the content of the participants' comments concerning typicality. Regarding dyadic coping as part of the partner's behavior, we formulated categories deductively based on the STM (Bodenmann et al., 2016). To assess inter-coder reliability, a trained graduate student coded data of *n* = 25 (21%) randomly selected couple material independently using the final coding system. Inter-coder reliability was 95.7% or on average κ = 0.96 (Rädiker and Kuckartz, 2019).

#### Global typicality categorization of participants at case level (scaling structuring)

In the second block, we used the deductive qualitative analyses method of scaling structuring following Mayring (2015) to categorize participants' view of the couple interaction in the lab on a case level as *typical, mixed/slightly typical*, or *untypical*. This categorization drew on the categories of the first analysis step (see above). For this part of the qualitative analyses, we formulated comprehensive definitions, anchor examples, and coding rules to enable unequivocal allocation of each of the participant's transcripts to one of the three deductive categories (typical, mixed/slightly typical, untypical). These coding rules were based on the multidimensional definitions of ecological validity by Schmuckler (2001) and Holleman et al. (2020) with a focus on the subjective experience of the partner behavior during the stress conversation. For each participant, all categories from the first analysis step were reviewed to reach a decision, whether this participant viewed the partner's behavior in the lab situation as typical, mixed/slightly typical, or untypical. A short description of the decision rules is given here:

- ***Typical*** partner behavior should appear natural or be generalisable to everyday life at home. Therefore, partner behavior should be coded as typical if the subject (a) identifies it as typical, characteristic or representative, (b) states that it occurs always, often, frequently, etc., or (c) that it occurs in this way at home or in everyday life or that it seemed natural.
- ***Slightly typical/mixed*** was coded when the participant described the partner behavior either (a) as slightly typical or sometimes typical, or (b) the participant comments on support behavior that was varying between situations (e.g., depending on the partner's stress level), or (c) ambivalent/contradictory statements (some aspects typical others untypical).
- Cases were rated as ***untypical***, if the participant describes the partner behavior as (a) untypical, uncharacteristic or unrepresentative, or (b) states that it never, hardly ever, rarely, etc. occurs in this way, or (c) does not occur like this at home or in everyday life or seems unnatural.

This coding rule was tested with a sub-sample of *n* = 25 couples (21%). This trial run lead to an adjustment of the slightly typical/mixed category (addition of variation between situations, see above). Additionally, minor changes were made to the wording of the typical and atypical categories to facilitate categorization. Additionally, we added the category ***Not classifiable*** for cases that did not touch on typicality in the lab situation or cases where the couples turned the intended DC conversation into a conflict conversation. All steps of the qualitative analyses coding process were supervised closely by the first author.

### Mixed-methods analyses

Due to the nominal scale level, non-parametric chi-square cross-tabulations were calculated to analyse the distribution of typicality by age and gender. First, we divided the sample into three age groups (< 45, 45–64, >65 years). Pearson chi-square tests were carried out with age and gender as dimensions using bootstrapping (5,000 samples). We excluded those individuals from the analysis that could not be classified in the qualitative analyses (*n* = 3 women, *n* = 6 men). We used IBM SPSS 29 for the analyses.

## Results

We will first present the results of the deductive scaling structuring followed by an in-depth description of the inductive categories regarding what participants found un-/typical regarding the partner's behavior, the couple conversation or the lab setting.

### Global typicality categorization of participants at case level (scaling structuring)

**Figure 1:**
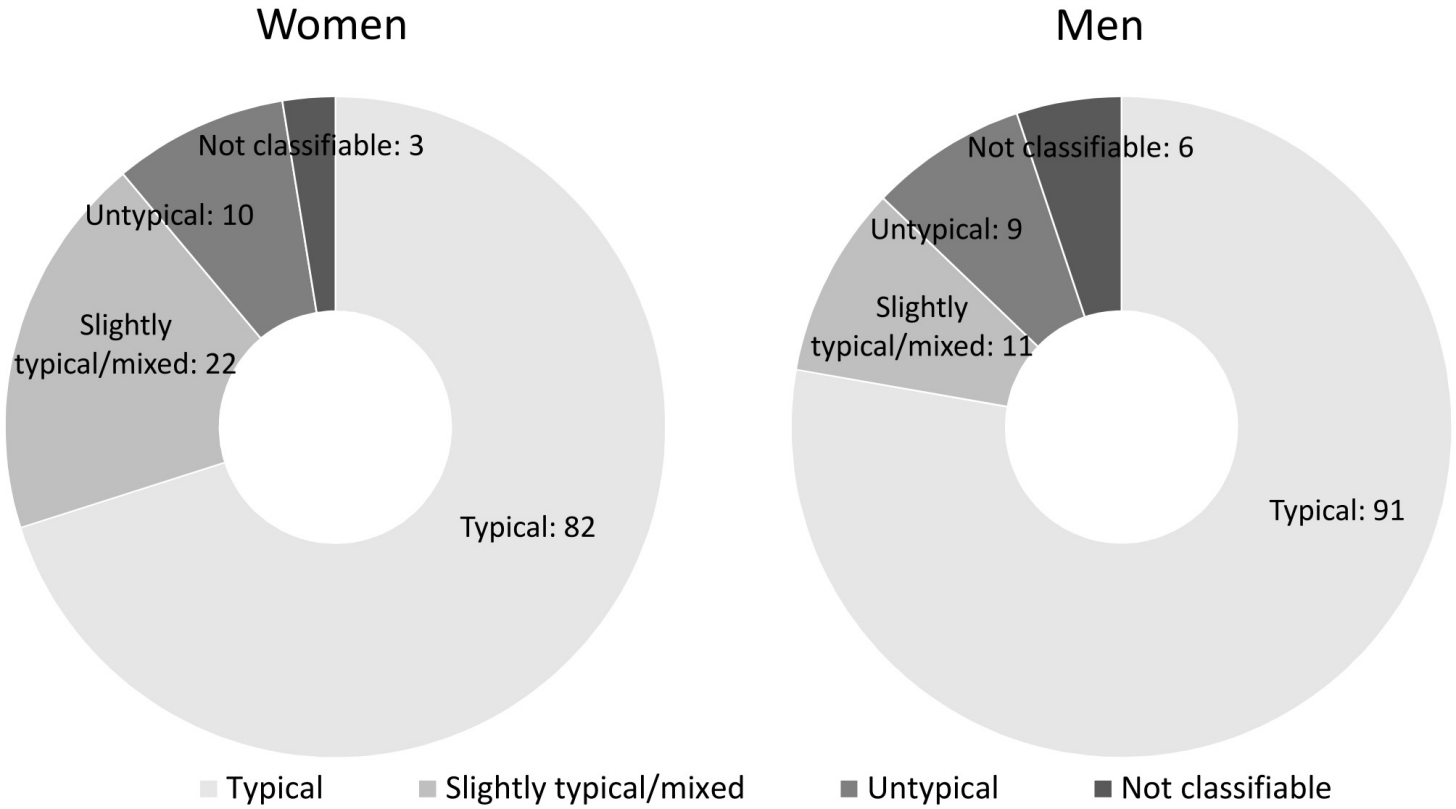
Structured scaling showing the absolute person frequency of women **(left)** and men **(right)** perceiving the partner's behavior in the stress conversation as typical, slightly typical/mixed, or untypical. *N* = 117 couples.

The scaling structuring showed that overall, 77.8% of men and 70.1% of women considered the partner's DC behavior during the stress conversation as typical, 9.4% of men and 18.8% of women as slightly typical/mixed, 7.8% of men and 8.6% of women as untypical. Of men, 5.1% could not be rated due to a lack of information and 2.6% of women (see Figure 1 for frequencies).

### Categorization of which aspects participants perceive as typical (inductive/deductive analysis)

Overall, in total 2,146 codes were assigned to events within the video review task regarding that participants commented on regarding the un-/typicality of the lab situation and the couple behavior. The final coding system covered the main categories *typical* (1,339 codes, 62.4%), *mixed/slightly typical* (121 codes in total, 5.6%)*, untypical* (667 codes, 31.1%), and *non-classifiable* (19 codes, 0.9%). We used the category *Non-classifiable* when participants did talk about typicality but the content was irrelevant to the research question, for instance talking about hypothetical alternative versions of the lab conversations or talking only about typical behavior at home. One participant did not touch on typicality at all.

For each of these main categories we formulated subcategories responding to the ecological validity criteria: *partner characteristics/partner support* (response), *conversation* (stimulus), and *setting (*context*)*. In the following sections, we present each main category (*Typical, Slightly typical/mixed*, and *Untypical*) in more detail and specifying the frequency of each sub-/category with the absolute person frequency (the number of participants talking about this topic) and the relative person frequency in percent. We present global comments first, followed by more specific categories which are presented in descending order of frequency.

#### Typical

Overall, 81.2% of participants (*n* = 190) mentioned something typical about their partner's behavior, while around two thirds of participants viewed an aspect of their partner's support as typical (63.2%, *n* = 148, see Table 2). About a third observed something typical about their stress conversation (*n* = 84, 35.9%), while none of the participants viewed anything about the lab setting itself as typical (*n* = 0, 0%).

**Table 2:** Factors judged as typical by participants regarding the partner or the conversation.

**Category Subcategory**	**Absolute topic frequency**	**Absolute person frequency**	**Relative person frequency (%)**
Partner characteristics	365	190	81.2
General	170	143	61.1
Non-verbal	44	34	14.5
Objective, analytical	20	16	6.8
Attentive	14	11	4.7
Calm, relaxed	14	12	5.1
Humorous	12	9	3.8
Thoughtful	12	11	4.7
Passive, restrained	11	9	3.8
Dominant	9	8	3.4
Accepting	7	6	2.6
Emotional	6	6	2.6
Lack of empathy	6	4	1.7
Restless, nervous	6	5	2.1
Various	34	32	13.7
Partner support	332^a^	148^a^	63.2^a^
General	40	37	15.8
Listening/paraphrasing/giving space	98	76	32.5
Emotion-focused DC	66	49	20.9
Problem-focused DC	44	36	15.4
Giving space	23	20	8.5
Specific personal aspects	22	19	8.1
Critical analysis	21	17	7.3
Asking questions	20	16	6.8
Conversation	124^a^	84^a^	35.9^a^
General	54	46	19.7
Topic, content, type of conversation	35	31	13.2
Conversation sequences	30	25	10.7
Setting	0	0	0
Total	821^a^	221^a^	94.4^a^

DC = dyadic coping. Omitted are categories with n < 5. ^a^Frequencies of main categories include frequencies of rare omitted subcategories.

#### Typical partner characteristics

Mostly, participants commented on a more general level that the partner behaved in typical and familiar ways similar to conversations happening in their daily lives (“basically, that's him,” 1,066, woman; *n* = 143, 61.1%).

About 15% of participants (*n* = 34) commented on non-verbal aspects of the partner's behavior. The accounts were filled with a sense of great familiarity, such as knowing the partner for years, their facial expressions and gestures, body posture, the attentive way of nodding while listening, eye contact or to a lesser degree the lack of eye contact. One woman observed their partner's fist resting on the armchair then moving in a specific way that was very familiar and telling of the partner's emotional condition:

He opened his fist and pressed a little with his finger. That's very typical. There's always a lot going on inside of him and then you can see it coming out in very small gestures. (No. 3033, woman)

Participants commented on a range of different ways the partner engaged in the conversation. Due to space restrictions, we will pick out some topics to illustrate what participants thought was typical during the lab situation. Overall, a sense of familiarity was visible, hinting to the authenticity of the lab conversations. For instance, participants commented on the way their partner was looking at things objectively and analytically, or being thoughtful and pragmatic. Some appreciated this level-headed perspective when they shared about their personal stress (“She is sensational, she can think fast.” No. 2069, man), for others this analytic approach was a source of distress:

It is sometimes too matter-of-fact for me and I almost cry because I think I'm a person of the heart and he's very much a person of the head. (No. 2071 woman)

Other participants mentioned the emotional or humorous way of the partner as typical for these kinds of conversations.

Sometimes he laughs in between and that often helps me to put my problems into perspective. (No. 1068, woman)

Some partner characteristics were viewed more negatively, like dominant behavior, showing a lack of empathy, the partner being passive or restrained in stress conversations or restless and nervous.

She constantly moves her hands a little and looks back and forth. Although, I have to say that this is perhaps something I know from home. (No. 3116, man)

#### Typical partner support

Partner support in the lab was often viewed as quite similar to the support experienced in the couple's daily life. Participants commented on this in general terms (*n* = 37, 15.8%) and described the support of their partner as “normal,” “commonplace,” or “familiar.” Some even said the support would always look like the support shown in the lab situation, as one participant explained:

That's my wife, typically my wife. She always tries to support me, both mentally and if I have a difficulty somewhere, then she definitely tries to provide me with immediate support. (No. 2069, man)

A third of participants (*n* = 76, 32.5%) commented on their partner's way of listening and their attentiveness, mostly regarding quantity (“listening for a while,” No. 1014 man) or quality (listening attentively), as one participant framed it:

What is perhaps typical is she listens in a very focussed and attentive way. (No. 1005, man)

Participants valued their partner's listening skills and having their partner's full attention when sharing about a problem and feeling heard. They felt they had the space to expand on their stress experience. Some participants explicitly mentioned their partner's ability to summarize or paraphrase what they understood:

It's also very typical what he does. He repeats what I say to him. He gives me the understanding that he has understood. But (laughs) he does that at home too. (No. 1046, woman)

About 20% of participants (*n* = 49) commented on typical emotion-focused DC within the lab situation, like feeling understood, taken seriously, or that their partner is genuinely interested and emphatic.

And above all, that she's interested. So, it's not just because we did this exercise there, because that was the task, but it's her nature. (No. 2087, woman)

Fifteen percent of participants (*n* = 36) mentioned their partner's problem-focused DC as typical. Here, participants commented on their partner's way of proposing solutions and giving advice. Those were mainly appreciated, for some, however, these suggested solutions were not what they needed in the moment or did not seem to fit:

And then he gives me tips and tells me what I have to do. And that's actually quite counterproductive at that moment (laughs). (No. 1046, woman)

Some accounts captured specific familiar ways of the partner's support (*n* = 19, 8.1%). Participants talked about how the course of the conversation was very familiar, almost knowing beforehand what their partner will suggest next, or having had very similar conversations before.

I know my wife's opinion and I wasn't actually surprised by the way she argued, because that's almost, yes, almost self-evident for me. The way she said it was nothing new for me in that sense. (No. 3106, man)

Some participants described their partner's way of critically challenging them as typical (*n* = 17, 7.3%). This was valued by some as it offers a new perspective or another opinion. Others experienced these challenges as criticism or described it as a double-edged sword:

It is also the case that she sometimes asks probing questions, in a malicious sense, but she can also challenge me from time to time and I'm actually happy to accept that. At least on hindsight (laughs). (No. 1005, man)

Additionally, participants viewed their partner's validation and help to find the right words to name the exact feelings as typical. About 7% (*n* = 16) viewed their partner's way of asking questions as typical, which was mostly valued and for some seemed almost a bit “too much” (No. 1044, woman).

#### Typical conversation

About a third of participants (*n* = 84) thought some aspect of the conversation was typical. The content or the way the conversation unfolded was generally viewed as representative of situations at home. They were labeled as a “habit,” “normal,” and a typical day-to-day sequence. The lab conversation seemed to follow a common sequence of back-and-forth conversation in familiar ways, for instance like a “ping pong game” (No. 1016, woman). Some mentioned that they had talked about the exact topic a couple of times before so there were not many surprises. One woman phrased it in the following way:

That could have been at home without the microphone. (No. 2002, woman)

### Slightly typical/mixed

Around 16% of participants (*n* = 38) mentioned aspects that were categorized as slightly typical/mixed (see Table 3). Here, participants only talked about the partner support and did not mention either characteristics of the setting or conversational aspects as being slightly typical or mixed.

**Table 3:** Factors judged as slightly typical/mixed and untypical by participants regarding the partner.

**Category Subcategory**	**Absolute topic frequency**	**Absolute person frequency**	**Relative person frequency (%)**
***Slightly typical/mixed***			
Partner characteristics	0	0	0
Partner support (situation-dependent)	58	38	16.2
Mood	15	12	5.1
Time	11	9	3.8
Topic	11	9	3.8
Opinion/perspective	10	8	3.4
Stress level	5	5	2.1
Interest/attention	5	4	1.7
Conversation	0	0	0
Setting	0	0	0
Total slightly typical/mixed	65^a^	42^a^	17.9^a^
***Untypical***			
Partner characteristics	118^a^	84^a^	35.9^a^
More attentive	27	25	10.7
Non-verbal	25	23	9.8
More passive, restrained	16	13	5.6
Less emotional	15	11	4.7
More patient	9	7	3.0
More calm, relaxed	7	7	3.0
Various	19	15	6.4
Partner support	97^a^	51^a^	21.8^a^
Listening more	36	24	10.3
Giving space more	33	23	9.8
Emotion-focused DC	14	11	4.7
Specific personal aspects	6	6	2.6
Asking questions	5	4	1.7
Conversation	88	61	26.1
General	8	8	3.4
Course of the conversation	48	32	13.7
Focussed conversation	16	16	6.8
Longer duration	11	8	3.4
Topic/content	5	5	2.1
Setting	109^a^	67^a^	28.6^a^
General	35	30	12.8
Time to deal with the topic	17	15	6.4
Being undisturbed	21	20	8.5
Separate seating arrangements	25	23	9.8
Physical distance	9	9	3.8
Total untypical	416^a^	142^a^	60.7^a^

DC = dyadic coping. Omitted are categories with n < 5. ^a^Frequencies of main categories include frequencies of rare omitted subcategories.

#### Partner support (situation-dependent)

Participants noted that the behavior exhibited by their partners in the lab conversation was typical only of certain situations at home. These circumstances included situations where both partners have the opportunity to talk without interruption, when the supporting partners are not experiencing stress, and are in the right mood (“it depends a bit on the time of the day or his mood,” No. 2043 woman). Participants also mentioned variability in the experienced typicality due to the topic of the conversation. Partners were seen as less engaging at home compared to the lab situation if they were not interested, the topic was of minor relevance, or they had talked about it too many times before.

But in other situations, he tends to withdraw. So, until I realize, “Oh, whoops, he's not even in the room.” However, when it really matters, he's there. (No. 3086, woman)

#### Untypical

Over 60% of participants (*n* = 142) mentioned something untypical about the lab conversation. About a third viewed certain aspects of their partner's behavior as untypical (*n* = 84, 35.9%), whereas around 22% of participants (*n* = 51) viewed an aspect of their partner's support as untypical (see Table 3). About a fourth observed something untypical about their stress conversation (*n* = 61, 26.1%), and 29% (*n* = 67) mentioned an aspect of the lab setting itself as being not typical.

#### Untypical partner characteristics

Some participants noted that getting their partner's full attention was untypical (*n* = 25, 10.7%). They viewed the situation as artificial. Usually, their partner would be multitasking, half-distracted by household chores, children, or electronic devices. Participants perceived their partners to have more eye contact while listening attentively. One woman phrased it like this:

And like I said, this is now an exceptional situation that he's sitting there like this, he cannot do anything else and has to listen to me, actually. (No. 3008, woman)

In line with this, partners were perceived as more patient (“In everyday life, she might have been quicker to tell me, ‘Hey, what do you want to say now?”' No. 2032 man).

Participants noticed their partner looking at the camera from time to time, which shows their partner's awareness of being filmed. Others viewed their partners as untypically restrained or calm in the lab situation. Usually, these partners were described as moving around more or raising their voice more at home but they seemed to be holding back in front of the camera:

I don't know if it's the camera or (laughs) usually it's a bit/a bit more heated. (No. 3078, woman)

Some partners were described as tense or even freezing up, as if they were trying to hold back their more unfiltered reactions. They seemed to monitor what and how they were saying things. Camera awareness seemingly led to some partners being less spontaneous or relaxed compared to their usual conversations at home. In some cases, the opposite was true, that partners behaved in a more relaxed way than at home.

It is not typical for her to be as relaxed as she is now. (No. 2014, man)

#### Untypical partner support

A few participants observed that their partner seemed to listen more during their lab conversation (*n* = 24, 10%). Participants were positively surprised about their partner letting them finish what they wanted to share without interrupting earlier. One participant half-jokingly compared the lab situation to a protected environment and as some kind of test that they wanted to pass by showing certain couple behaviors. He implied that giving each other space would be the preferred way of interacting:

Yes, in an unprotected situation we would probably have talked over each other a few times, both of us. So, we've been good now/today, haven't we? We made sure that we didn't fail the test, that we passed it, because you're not supposed to interrupt each other. (No. 2115, man)

Some participants perceived their partners as showing more effort in being helpful, being more engaged and displaying more interest. One woman said about her partner:

But now, in this study in which we are participating, it seemed to me that he was of course obliged to say a few words and sentences on the subject. (No. 3057, woman)

#### Untypical conversation

Regarding their conversation, about a fourth of participants commented on aspects of the lab conversation that they deemed untypical (*n* = 61, 26.1%). For some participants, it was uncommon to have the same amount of time and focus for the conversation available compared to at home, where they would be multitasking or interrupted regularly by the phone or their children.

That we have the concentration for it [the conversation] that the setting was in a way that we could really sit together in peace (laughs) and talk without the children interrupting or fighting and distracting us or anything like that. (No. 1096, man)

In some of the accounts, it was noticeable that the lab setting seemed to motivate them to show “exemplary behavior” (No. 2049, man). It was mentioned that they tried not to blame each other or they were steering the conversation toward less divisive topics or were more controlled. Compared to the lab setting, couples would show an increase in volume and pitch more often at home, which they seemed to hold back in the lab. Others described the lab conversation as calmer and more relaxed, less emotional (“in a positive and in a negative sense,” No. 2111, man), more harmonious, or even boring. Also, the choice of words seemed untypical for some. For example, the tone was more casual or they used more swear words (No. 2068, man). One participant who described their conversations at home as “more childlike,” also observed a change across the couple interactions in the lab, from the two dyadic coping interactions to the third interaction which was a conflict conversation. Specifically, this participant described how they adapted to the lab setting over time and their conversation got more comparable to their day-to-day interactions:

You realize “Okay, we're being filmed,” although that has subsided, especially in the second and third conversation. (No. 1083, man)

For some, the comparison between the lab situation and their conversations at home led to a level of reflection about how their ideal conversations could look like and the discrepancy to conversations at home. Perhaps as if the lab situation showed them how they were able to talk constructively about a topic without being distracted or stressed. One woman jokingly thought about recreating the lab situation at home, because she enjoyed the attentiveness of her partner and the conversation.

Yes, I wish we could talk with each other like that at home. I think I'll set up a camera at the dining table now. When my husband sees a camera, he's completely different. (No. 2054, woman)

#### Untypical setting

About a third of participants commented on the untypical setting of the lab conversation (*n* = 67, 28.6%). They mainly mentioned the untypical seating arrangements with two separate armchairs (“If such a conversation had taken place at home, I would probably have been lying in his arms,” No. 1051, woman) or the focused and uninterrupted time they had in this quiet room for a set amount of time. General comments captured the artificial, encapsulated, or “synthetic” nature of the lab setting. One participant, who seemed distracted by the cameras, labeled the lab situation as an “experiment” (No. 2106, man). Some participants mentioned the feeling that their conversations were taking place more casually at home, thus feeling livelier. As one woman described it:

Sometimes I lean against a doorframe and he sits at his computer. Then we talk like that for a moment—it's a bit random sometimes. (No. 3113, woman)

### Mixed methods results

Pearson chi-square tests were carried out with age and gender as dimensions regarding the typicality of the lab setting. The global chi-square test was significant (χ^2^ = 11.700; *p* ≤ 0.020). Results differed with regards to gender. For men, the test was significant (χ^2^ = 11.837; *p* ≤ 0.019), while for women, it was not (χ^2^ = 5.689; *p* ≤ 0.224). On a descriptive level, younger participants tended to view their partner's behavior as more typical, while untypical and slightly typical/mixed occurred more in the older age groups. This distribution was more pronounced for men then for women. For a contingency table see Supplementary Table S1.

## Discussion

Little is known about the ecological validity of couples' behavioral observations in a laboratory setting. The current study aimed to ask study participants themselves what they viewed as un-/typical about the lab situation and their partner's behavior compared to their usual situation at home. The results of this inductive analysis suggest that more than half of the participants viewed their partner's behavior as typical.

In particular, participants viewed their partner's listening, emotion- and problem-oriented DC and their general behavior as quite typical, which is comparable to the ecological validity dimension of *response* (Schmuckler, 2001). Whereas, the couples were less likely to describe aspects of the conversation as typical, comparable with the *stimulus*. Only certain aspects of the conversation were regarded as typical, such as the topic of conversation, or the course of the conversation. In sum, these results indicate that the behavioral observation in the laboratory did not have a strong influence on the way couples interacted with one another. As expected, none of the participants mentioned any aspect of the specific laboratory setting as typical, the *setting* dimension of ecological validity. Despite laboratory conditions, which are completely different from a conversation at home, we nevertheless find similarities in DC across the two situations, both in terms of behavior, topics, content as well as the course of the conversation. This may be interpreted as an indicator for the multidimensionality of ecological validity.

Participants commented on several aspects of the lab conversation as slightly typical or mixed. Here, partner-related factors were predominantly mentioned. It is interesting to note that the support of the partner was not perceived as atypical *per se*, but was contextualized by comparing it to the situational variability at home. The extent of the deviation frequently depended on the partner's mood or stress level, the timing of the conversation, the topic, the opinion or perspective of the partner. It seemed as if a certain variability in DC conversations and partner behaviors were viewed as normal in the everyday lives of couples. Along this line, conversations in the lab seemed to be most comparable to home situations when the couple was undisturbed, not particularly stressed, focused and calm. These factors align with STM theory (Bodenmann, 1995, 1997, 2000), which suggests that DC depends on coping resources, temporal aspects, and motivation, thus covering the abovementioned variability in daily DC interactions. Furthermore, there was noticeable variation among participants in how they perceived their partner's support, with some finding it more inconsistent than others. This points to a potential lack of ecological validity for at least some of the observed couple interactions. One solution as suggested by Foster et al. (1997) would be the exclusion of “invalid samples” based on their ratings of the typicality. Another solution would include a better understanding of which situations at home the lab situation is similar to.

Examining of the untypical aspects mentioned by participants, it seemed that the lab situation created an artificial bubble with little distraction, which likely promoted a calm and concentrated atmosphere. Only some participants felt actively distracted by the camera. However, the spatial separation caused by the physical distance of the two armchairs placed in the examination room was viewed as irritating and untypical by some participants. To summarize, the atypical factors of the partners, the conversation, or the situation described can influence interactions in the laboratory in different ways. Participants in the current study regarded the lab situation as more typical compared to the participants in the at-home video-taped conversation situations in the study by Foster et al. (1997). This is somewhat surprising since data collection took place in the couples' homes and it may be assumed that participants would regard the conversation at home as more typical than the unfamiliar lab setting. One explanation for this unexpected finding may be the conversation topic that couples talked about during the observation sequence. In our study, couples mentioned that they had talked about the specific topic they had chosen before and thus were already familiar with their own and the partner's views. The sample by Foster et al. (1997), however, talked about the recent loss of employment, thus a new and potentially emotional topic with major implications for the couple. This different stimulus may have impacted on the typicality ratings.

Some participants found the lab situation artificial because, unlike the controlled scenario, in everyday life, it is common for both partners to experience stress, not just one. The situation where one person can simply talk while the other listens does not necessarily correspond to reality. This implies that the lab setting is limited to replicating one of the two primary real-life scenarios, which is still somewhat ecologically valid: one partner is stressed while the other is not. The data suggest that the lab setting accurately reflects this particular scenario. However, the lab environment did not effectively mimic situations where both partners are experiencing stress.

Interestingly, ecological validity may vary depending on what type of couple interaction participants were comparing the lab setting to. At home, various influencing, situational or motivational factors are at play during couple interactions. It appeared that usually, couples would have time for shorter interactions only, while multitasking simultaneously or being interrupted by smartphones (“technoference”; McDaniel and Coyne, 2016, p. 85). Thus, for these kinds of situations, the ecological validity of the lab setting may be quite low. If undisturbed, however, conversations at home could follow similar patterns to those in a lab setting. The ‘real world' (Hammond, 1998) appears to be a heterogeneous set of interaction situations in a couple's life.

We did not assess the motivational aspects behind participants' behavior, like self-presentation or social desirability biases (Paulhus, 2001), which may have influenced the assessment. Some participants observed that their partner was looking at the camera, was distracted by the cameras or would have behaved differently, if no cameras were present. Thus, some biases may indeed influence video-taped couple interactions in the lab.

The gender and age differences discovered in the present study could indicate a complex interplay at the three levels of ecological validity. The lab situation was experienced as more artificial by older participants, while younger couples may have been more used to having technical equipment as part of their lives than older couples (OECD, 2020), thus, they adapted to the artificial setting more easily.

### Strengths and limitations

A major strength of the study is the focus on the participants' views on behavioral observations in a laboratory setting. To our knowledge this study design is the first of its kind and highly relevant to the field of behavioral couple observations. Further, we followed the applied qualitative content analysis in a transparent, rigorous, and rule-oriented fashion. To counteract the subjective nature of the qualitative analyses, we ensured that each step was carefully supervised and inter-coder reliability was good. Caution is required when interpreting our categorizations of typicality since we did not ask explicitly for a general rating. Thus, it is not possible to determine how typical the interviewed couples perceived the lab conversation overall.

We did not assess how participants generated their own internal template of *typical day-to-day partner behavior* against which to compare their partner's behaviors in the review situation. The implicit assumption of a possible pre-existing internal template was made and could easily be contested, for instance in terms of its fluidity or context dependency. At the very least, this adds another layer of complexity to the current analyses.

Regarding the sample, one needs to be mindful that the couples were in a long-term relationship with prior experience in the laboratory setting, thus reducing novelty effects and perhaps influencing spontaneity. A small number of participants indeed commented on this fact, which may indicate a certain habituation to the lab setting. It would therefore be of interest to repeat this study with a sample without prior lab experience to see whether we overestimated the ecological validity.

As is often the case, the current sample was quite homogenous and consisted of mostly educated cohabiting heterosexual couples with high degrees of relationship satisfaction. Due to the nature of the longitudinal study and data collection for this particular analysis at wave t10, couples were in long-term relationships. Partners in long-term relationships know each other very well and may have quite ingrained patterns of interaction. Therefore, this sample may be particularly suitable at assessing whether a particular partner behavior is typical for everyday life or not. However, the findings may not be generalizable for couples in short-term relationships who are not cohabiting. An additional factor affecting generalizability may be the fact that couples willing to participate in dyadic research may not be representative of the whole population (Barton et al., 2020). Future research should aim to include samples more heterogeneous regarding relationship satisfaction, educational level, sexual orientation, or non-WEIRD samples (Western, Educated, Industrialized, Rich, and Democratic; Henrich et al., 2010). The artificial laboratory setting might be perceived differently by marginalized groups who may be more mistrustful when interacting with the dominant culture. Moreover, video recording is more common in some cultures than in others, plus, some groups may have a more reserved attitude toward scientific studies, potentially leading them to behave less naturally in such settings. Therefore, it would be valuable to conduct similar studies in other cultural contexts to assess generalizability.

Additionally, future research should consider methodological triangulation, to assess ecological validity in more detail and more objectively, for example, by comparing field and lab observations directly or use brief quantitative typicality ratings or diary data. Another line of future research could focus on the typicality of the participant's own behavior. In the current study, we asked participants to assess only their partner's behavior for typicality; however, subjective reflection and assessment of one's own interaction behavior would be an equally valuable option. Moreover, in terms of second-order cybernetics (Luhmann, 2021), it should be noted that participants commenting on their partners are not neutral; their assessments emerge from their own expectations, prior experiences, and the laboratory context. In future studies, it may be valuable to address this meta-level and, for example, assess the self-reflexivity among study participants regarding how their perspectives, preconceptions, and the artificial setting of the video-review task, together with the video perspective on the partner, structure their evaluations.

### Conclusions and implications

To conclude, partner behavior was viewed as typical by the majority of participants. This points to a considerable degree of ecological validity for DC studies conducted in the laboratory and grounds behavioral couple observations on a firmer footing. At the same time, however, multidimensionality regarding the setting, conversation, or the partner could reduce overall ecological validity. Particularly, younger couples with children in the household (or couples with other care tasks) may have shorter conversations with more interruptions while multitasking simultaneously. The findings point to the high ecological validity of stress conversations in a lab setting only for certain conversations at home where both partners have the time to talk without interruption and when the supporting partners are not stressed themselves and are in the right mood.

An important implication for couple research is that laboratory studies with behavioral observation still seem to be a valid way of investigating communication patterns in couples (DC and conflict). Although the idea of passively and continuously collecting real life data via smartphone sensors is tempting, it is very costly overall (in terms of personnel and finances), ethically not without its challenges, as it directly intervenes in the lifeworld of the couples, and also relies on machine learning to extract the interesting interaction sequences from the large amount of data (Keusch and Conrad, 2022). The fact that our study shows that we have a relatively high ecological validity with the design used so far is encouraging against this background and proves that conscientious research can continue to be conducted with this approach now with the solid findings of the current study backing the assumption of sufficient ecological validity. An implication for relationship programs seems to be the relevance to address potential detrimental effects of technoference on couple communication and relationship satisfaction (Mushquash et al., 2022) while promoting undisturbed time set aside for DC as well as conflict conversations.

## Data Availability

The datasets presented in this article are not readily available because the sensitive data will not be shared publicly. Requests to access the datasets should be directed to Guy Bodenmann, guy.bodenmann@psychologie.uzh.ch.

## Footnotes

1 Kerkhoff, D., Weitkamp, K., Bernecker, K., Brandstätter, V., Dolcetti, M., Landolt, S. A., et al. (submitted). Stability and change in relationship quality: a 10-year dyadic study.
