# Supplementary material for: “That's just typical!” A qualitative video review study on the ecological validity of couples' stress conversations in the lab
Source: Front Psychol. 2025 Dec 2;16:1497690. doi: 10.3389/fpsyg.2025.1497690 (PMC12706654; doi:10.3389/fpsyg.2025.1497690)
Supplement: Supplementary file 1 [file Table_1.pdf]

Supplement Table S1

Contingency table of typicality for gender and age groups (≤44, 45-64, ≥65 years)

	Typicality					
Gender<br>Age groups (years)	Untypical	Slightly typical/mixed	Typical	Total	χ^2	*p*
Men						
≤44	0	0	23	23		
45-64	8	5	38	51		
≥65	1	6	30	37		
Total	9	11	91	111	11.837	.019
Women						
≤44	0	3	22	25		
45-64	5	11	38	54		
≥65	5	8	22	35		
Total	10	21	82	114	5.689	.224
Whole sample						
≤44	0	3	45	48		
45-64	13	16	76	105		
≥65	6	14	52	72		
Total	19	33	173	225	11.700	.020

Note. *n*=225; individuals that were not classifiable were excluded from this analysis (*n* = 3 women, *n* = 6 men).
